# Computer based method for identification of fibrotic scars from electrograms and local activation times on the epi- and endocardial surfaces of the ventricles

**DOI:** 10.1371/journal.pone.0300978

**Published:** 2024-04-16

**Authors:** Arstanbek Okenov, Timur Nezlobinsky, Katja Zeppenfeld, Nele Vandersickel, Alexander V. Panfilov

**Affiliations:** 1 Department of Physics and Astronomy, Ghent University, Gent, Belgium; 2 Department of Cardiology, Leiden University Medical Centre, Leiden, The Netherlands; University of Minnesota, UNITED STATES

## Abstract

Cardiac fibrosis stands as one of the most critical conditions leading to lethal cardiac arrhythmias. Identifying the precise location of cardiac fibrosis is crucial for planning clinical interventions in patients with various forms of ventricular and atrial arrhythmias. As fibrosis impedes and alters the path of electrical waves, detecting fibrosis in the heart can be achieved through analyzing electrical signals recorded from its surface. In current clinical practices, it has become feasible to record electrical activity from both the endocardial and epicardial surfaces of the heart. This paper presents a computational method for reconstructing 3D fibrosis using unipolar electrograms obtained from both surfaces of the ventricles. The proposed method calculates the percentage of fibrosis in various ventricular segments by analyzing the local activation times and peak-to-peak amplitudes of the electrograms. Initially, the method was tested using simulated data representing idealized fibrosis in a heart segment; subsequently, it was validated in the left ventricle with fibrosis obtained from a patient with nonischemic cardiomyopathy. The method successfully determined the location and extent of fibrosis in 204 segments of the left ventricle model with an average error of 0.0±4.3% (N = 204). Moreover, the method effectively detected fibrotic scars in the mid-myocardial region, a region known to present challenges in accurate detection using electrogram amplitude as the primary criterion.

## Introduction

Cardiac fibrosis involves an abnormal proliferation of connective tissue due to excessive deposition of extracellular matrix and has traditionally been associated with cardiac arrhythmias and dysfunctions [1–5]. Ablation, a technique used to treat arrhythmias, involves creating scars in diseased cardiac tissue to disrupt the abnormal electrical signals that cause arrhythmias [6]. Identifying targets for ablation often entails locating the regions responsible for initiating and maintaining arrhythmias. Multiple studies [7–10] have demonstrated that these regions are typically found within cardiac fibrosis areas, which are associated with the local spatial distribution of fibrotic tissue. Thus, determining the location and properties of fibrosis remains an essential area of research.

A gold-standard technique for identifying fibrosis within the heart is late gadolinium-enhanced (LGE) cardiac magnetic resonance (CMR) imaging [11–13]. LGE-CMR imaging relies on reference values—either dense fibrosis or healthy myocardial areas—to accurately estimate fibrosis density from the signal intensity of the LGE-CMR. However, these reference values are not always available. Histological research [14] suggests that hearts with non-ischemic cardiomyopathy (NICM) rarely contain areas of compact fibrosis, and areas deemed “healthy” myocardium may have varying degrees of fibrosis. In a study involving young patients by Haissaguerre et al. [15], it was found that cardiac arrhythmias can arise in hearts with no apparent structural anomalies on MRI scans. Instead, these abnormalities can only be detected through electrical recordings taken from the surface of the heart.

Electrical signal recordings are typically obtained from multiple locations on the inner (endocardial) surface of the heart during invasive clinical ablation procedures. Recent advancements have also enabled signal recording from the outer (epicardial) surface [16, 17]. Since the normal cardiac excitation wave propagates from the endocardial to the epicardial surface, epicardial intracardiac electrograms (EGMs) may contain significant information not only about epicardial fibrosis close to the electrode but also about fibrosis within the myocardial wall. Two sets of parameters are particularly important for extracting information about fibrosis from multiple EGMs. Firstly, computational studies have demonstrated that local activation times (LAT) can help in reconstructing areas of slow conduction on the endocardial surface of the heart using adaptive algorithms [18, 19]. Secondly, peak-to-peak amplitudes (PtP) are crucial parameters, as regions with lower amplitudes are associated with fibrotic areas near the recording electrode [14, 20–22]. In this study, we propose an approach to estimate fibrosis that combines information obtained from these two sets: LAT and PtP. To achieve this, we develop a cardiac model incorporating different levels of fibrosis, including models where fibrosis is induced in one of the ventricle segments located at the endocardium, mid-myocardium, and epicardium. Additionally, we create a model based on a detailed histological analysis of a patient’s heart with NICM. We compute the two sets of parameters from the recorded EGMs using both endo- and epicardial EGMs and apply optimization methods to predict the levels of fibrosis. We demonstrate that this method enables accurate estimation of the degree of fibrosis in the left ventricle sub-endocardial, mid-myocardial, and sub-epicardial segments by iteratively fitting the PtP and LAT of the unipolar EGMs.

## Methods

### Anatomical mesh generation including fibrotic tissue

We generated five distinct distributions of fibrosis for testing our algorithm. Four of these distributions were synthetically generated for a segment of the LV, while the fifth distribution was derived from histological measurements and includes fibrosis across the entire LV. In this section, we will present the details of how we generated the five different meshes.

#### Anatomical mesh

The anatomical 3D LV was extracted from one of the publicly available four-chamber heart meshes [23]. This dataset comprises finite element meshes generated from end-diastolic computer tomography scans. Ventricle fibers were generated using a rule-based algorithm [24]. Additionally, the mesh includes labels for each part of the heart. We modified this initial mesh to create a finite difference mesh. Firstly, the LV was manually rotated to align the base parallel to the XY plane. Secondly, the rotated mesh was positioned within a cube measuring 256 × 256 × 256. Thirdly, all cube nodes within the finite elements were labeled as myocardium. Finally, the fibers were approximated using the nearest four fibers from the initial mesh.

#### Fibrosis modeling in a given region

Fibrosis was simulated by designating a certain percentage of nodes as non-conductive within a specified region. For each mesh node, predefined density values were compared with uniformly distributed random values ranging from 0 to 1. If the density value surpassed the random value, the node was categorized as non-conductive, following a similar approach as described in Nezlobinsky et al. [25]. Below, we elaborate on the models we utilized for fibrosis distribution.

#### Four different artificial models of fibrosis

**Fig 1:**
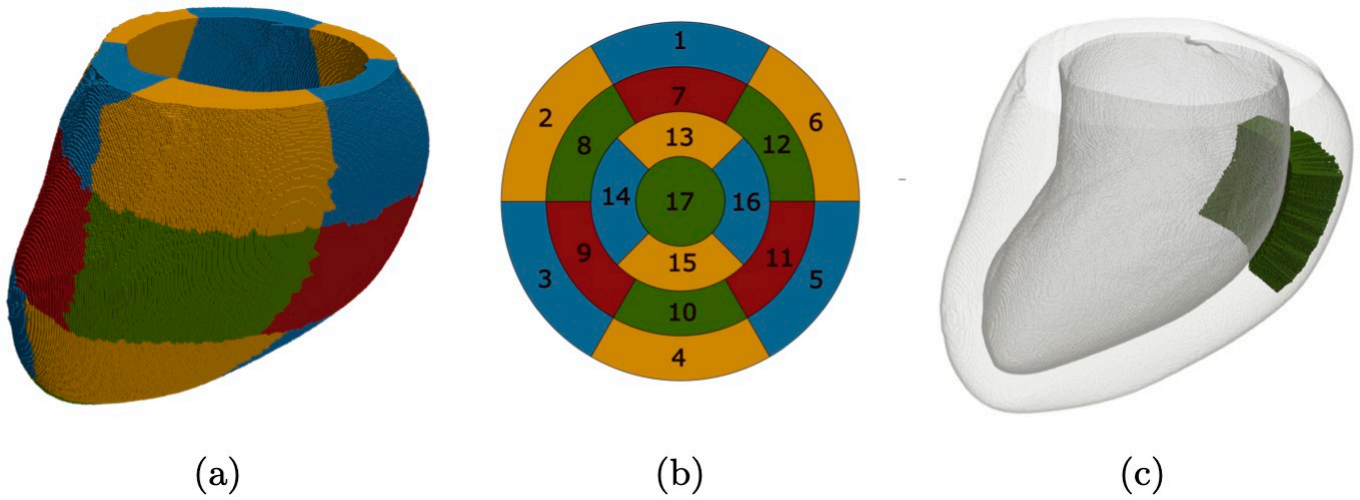
The LV segmentation model. (a) An approximate segmentation of the LV based on the 17-segment model. (b) A schematic diagram of the 17-segment LV model. (c) The mid-anterolateral segment (No. 12) used for evaluating the algorithm’s performance across four fibrosis patterns.

We constructed four distinct artificial models of fibrotic tissue to assess the algorithm’s performance. The original LV mesh was divided into 17 segments (see Fig 1a and 1b) using the cardiac segmentation guidelines provided by the American Heart Association [26]. To test our algorithm, we chose segment No. 12, specifically the mid-anterolateral segment (Fig 1c), and introduced fibrosis into this area.

**Fig 2:**
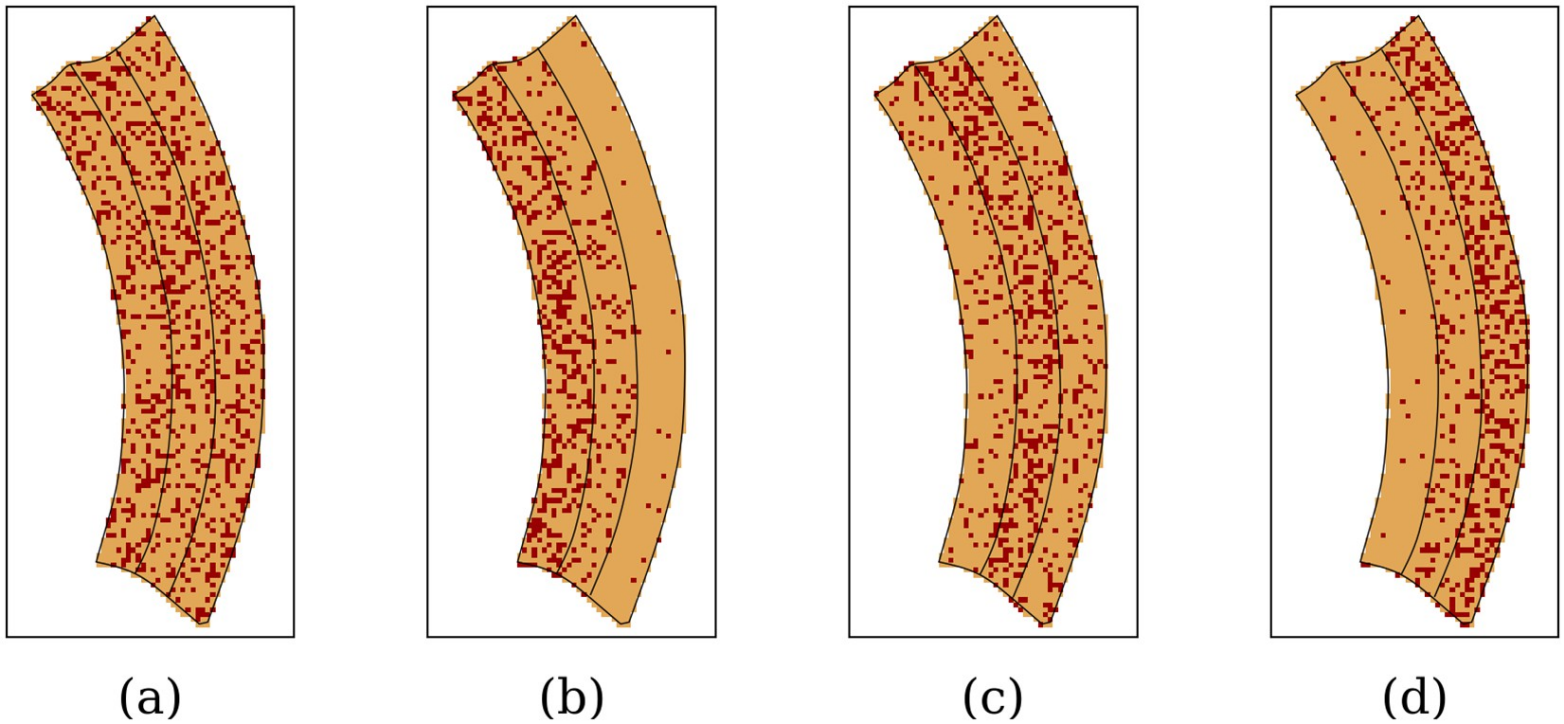
XY slice of the segment No. 12 with fibrosis patterns. (a) Transmural pattern. (b) Sub-endocardial pattern. (c) Mid-myocardial pattern. (d) Sub-epicardial pattern.

We opted to vary the location of fibrosis within the myocardial wall (see Fig 2 and Table 1): homogeneous transmural (panel a), sub-endocardial (panel b), mid-myocardial (panel c), and sub-epicardial (panel d). Firstly, for the transmural pattern, we set the fibrosis level to 25% and evenly distributed it across the entire segment. Secondly, in the sub-endocardial setting, we divided the segment into three layers and assigned varying fibrosis levels: 35% for the sub-endocardial layer, 15% for the mid-myocardial layer, and 2% for the sub-epicardial layer, situating the fibrosis primarily in the sub-endocardial layer. Thirdly, we opted for a setting with fibrosis concentrated mainly in the mid-myocardial layer (35% fibrosis), while the sub-endocardial and sub-epicardial layers contained only 10% and 15% fibrosis, respectively. Fourthly, the sub-epicardial setting is the reverse of the sub-endocardial setting. These four LV models with artificial fibrosis distributions will be employed to test our algorithm.

**Table 1:** Distribution of fibrosis within the segment No. 12 for various patterns.

Patterns	Location in the heart wall		
	Sub-endocard	Mid-myocard	Sub-epicard
Transmural	25%	25%	25%
Sub-endocardial	35%	15%	2%
Mid-myocardial	10%	35%	15%
Sub-epicardial	2%	15%	35%

#### Accurate model of fibrosis generated from histology measurements

We also constructed a fifth LV model featuring fibrosis patterns generated from experimental histology measurements of the heart. These measurements were acquired post-mortem from a patient with NICM [14]. The authors obtained transmural slices with a thickness of 5 mm and captured high-resolution microscopy images at a resolution of 10 microns. Additionally, the images were stained red to highlight fibrosis and yellow to delineate the myocardium.

The fibrosis in our model was generated based on 20 high-resolution images of the LV in the following manner.

In the first step, 2D density maps were computed from histological images. Firstly, the endocardial and epicardial edges were labeled using cubic splines. Secondly, the area between these edges was segmented into 72 × 6 parts, with 72 sections along the edges and 6 across. Thirdly, the fibrosis density in each segment was calculated as the ratio of red pixels, indicating fibrosis, to the total number of pixels, representing both fibrosis and myocardium.

**Fig 3:**
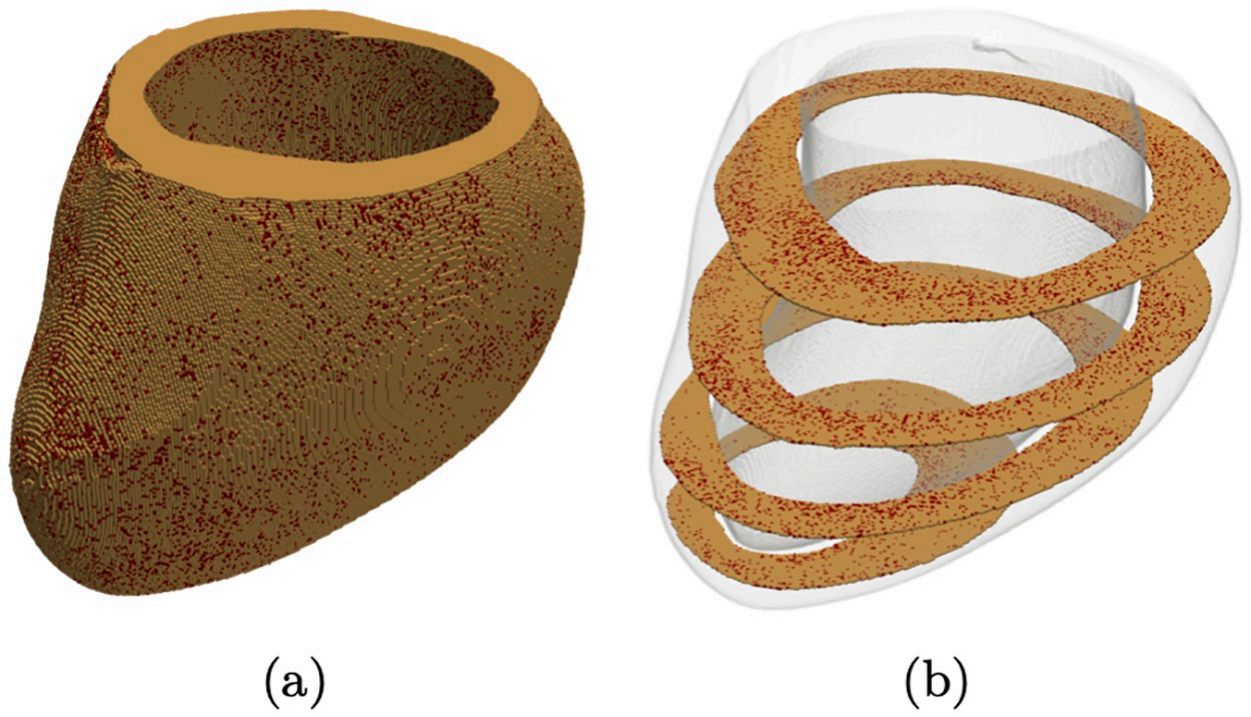
Fibrosis generated from histological images. (a) The LV with fibrosis. (b) Examples of slices generated directly from histological images.

In the second step, 3D fibrosis was generated in the LV mesh based on an interpolated set of 2D maps. Firstly, each XY slice of the 3D mesh was segmented into 72 × 6 parts. Secondly, 20 slices were selected to correspond to the histological images, so their fibrosis density was directly taken from the 2D histological set. Thirdly, the density of the slices in-between was interpolated using the available data: cubic splines were employed to interpolate the values along segments with the same label from different slices. Fourthly, the fibrosis density at each mesh point was averaged with the nearest values to yield a smoother map. Finally, by treating the fibrosis density as a probability value, the corresponding nodes were designated as non-conductive nodes. The final LV model with generated fibrosis is illustrated in Fig 3.

### Simulations on the mesh

We reintegrated the four synthesized segments into the original LV, whereas the fifth mesh already represented a complete LV.

For all five meshes, we replicated the sinus rhythm by simultaneously stimulating the endocardium of the LV [27]. These measurements were utilized to test our algorithm.

To conduct these simulations, we employed the Aliev-Panfilov cardiac model [28]. This model comprises two partial differential equations representing the monodomain description of cardiac tissue [29]:

$$\begin{array}{c} \frac{\partial u}{\partial t}=\nabla D\nabla u-ku\left(u-a\right)\left(u-1\right)-uv,\\ \frac{\partial v}{\partial t}=-\left(\epsilon +\frac{\mu_{1}v}{u+\mu_{2}}\right)\left(v+ku\left(u-a-1\right)\right), \end{array}$$ (1)

where *u* is the transmembrane voltage, *D* the diffusion tensor, *k* = 8, *a* = 0.1, *eps* = 0.01, *μ*_1_ = 0.2 and *μ*_2_ = 0.3. Numerical computations were performed using the explicit finite-difference method with time step *dt* = 0.0015 model units and space step *dr* = 0.1 model units. The 19-point asymmetric stencil was implemented for evaluating the Laplacian Eq 1 in an anisotropic medium [30]. Fibrosis and boundaries were treated by using the no flux conditions ($\vec{n}\nabla u=0$).

Additionally, we conducted tests to assess if we could induce an arrhythmia in the fifth mesh. For this purpose, we applied a series of 10 stimuli with a step of 28.5 time units to the apex region of the endocardium. This stimulation entailed a current of 100 units and lasted for a duration of 0.2 time units.

### Electrogram model

Throughout the simulations, unipolar EGMs were consistently acquired from both the endocardial and epicardial surfaces of the left ventricle, as they are part of the algorithm’s required input. This section will comprehensively detail the process of computing the EGMs.

The unipolar pseudo-EGMs were computed as potentials generated by current sources at a distance *r* from electrodes [31, 32]:

$$\begin{array}{c} EGM=\sum_{i}^{N}z\left(r_{i}\right)I_{i}, \end{array}$$ (2)

where *z*(*r*) is the transfer function that is inversely proportional to the power of two of the distance *r*, *I* is transmembrane current that is equal to diffusion part of Eq 1 and *N* is number of myocardium cells.

For the first four models, we only selected the 72 endocardial electrodes and 81 epicardial electrodes close to the segment of interest. However, for our complete LV model (fifth model), we computed 465 endocardial and 566 epicardial surface electrodes as input for our algorithm. These electrodes were distributed randomly across the surfaces of all segments.

The LAT was determined as the steepest downstroke of the unipolar electrogram. We used endocardial stimulation time as a reference for measuring LAT. Thus, in the case of sinus rhythm, endocardial LATs were always 0, while epicardial LATs were equal to propagation times (therefore, further references to LAT pertain to epicardial LAT). LATs contain information about fibrosis because the wave propagation speed depends on the presence of fibrosis.

For each signal, the PtP was calculated as the difference between the maximum and minimum values of the signal. In cardiac electrophysiology, both unipolar and bipolar EGMs are commonly used as markers for fibrosis [33]. From a physical perspective, unipolar EGMs are more sensitive to the remote myocardium since the influence of remote activation decreases as the square of the distance for unipolar EGMs, as opposed to the cubic distance for bipolar EGMs. Therefore, we assumed that unipolar PtP is superior for detecting fibrosis within the wall.

The optimization algorithm necessitates data on LAT and PtP for all surface points of the heart (over 100,000 for the endocardium, over 150,000 for the epicardium). Hence, we interpolated the electrode measurements into whole surface points using the inverse distance weighting method. This method computes scalar values between points as a weighted average of the available values within a chosen radius *R*:

$$\begin{array}{c} w_{i}={\left(\frac{max(0,R-d\left(x,x_{i}\right))}{R\cdot d(x,x_{i})}\right)}^{2}, \end{array}$$ (3)

where *d*(*x*, *x*_*i*_) is the distance between *x*, for which we need to obtain the LAT and PtP and *x*_*i*_ for which the value is known.

### Optimization algorithm

We utilized the LAT and PtP measurements from the sinus rhythm simulation on the LV containing the target fibrosis distribution as the target values for minimization. We will outline the pipeline of how we optimized the fibrosis level of the different segments in the LV. Within this pipeline, the same minimization algorithm was employed, which is explained in the second part of this section.

For the four LV models with fibrosis present in a single segment, our optimization process began by adjusting LAT values on the surface of the segment, thereby skipping the first two steps of the four steps described below. For the fifth model, we utilized the complete pipeline outlined below.

#### Optimization of the different segments

**Fig 4:**
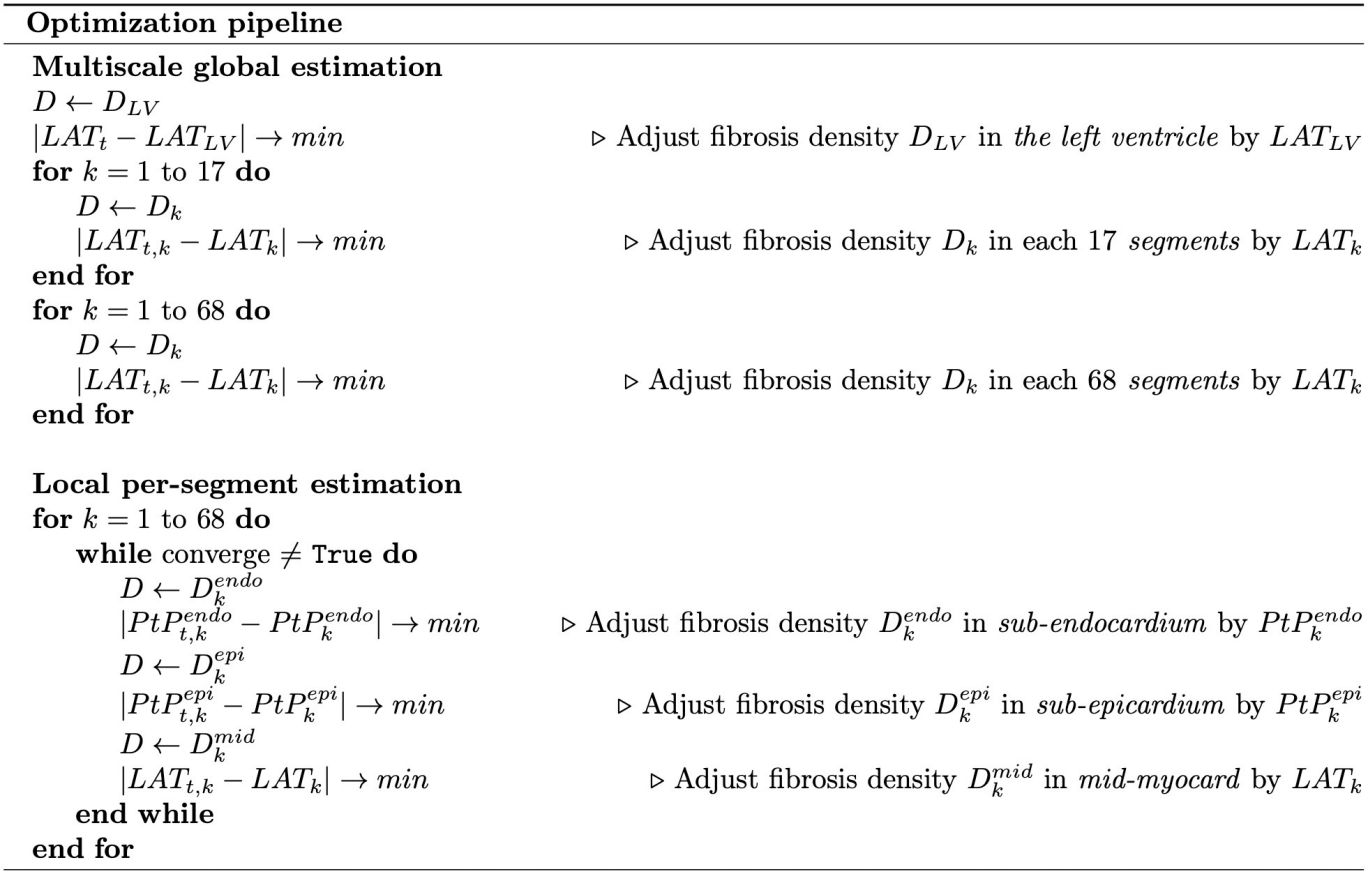
Optimization pipeline.

All optimization steps are outlined in the pipeline, as depicted in Fig 4, which we will elucidate step by step.

**Fig 5:**
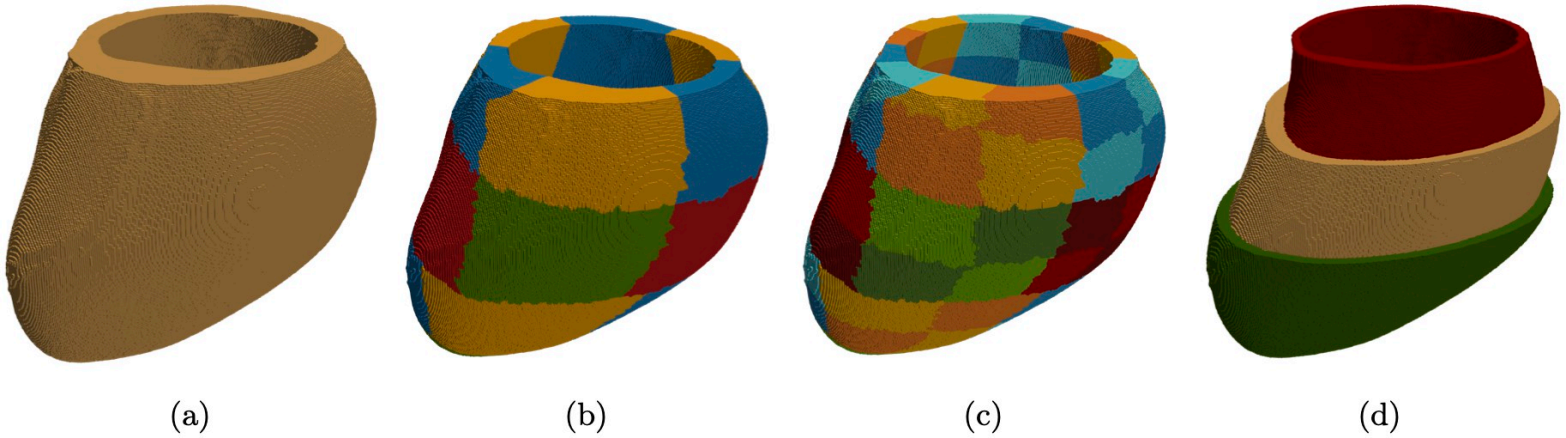
The segmentation process of the anatomical model of the left ventricle, employed for fibrosis optimization. (a) The LV used for the global optimization of step 1. (b) The 17 sections utilized in step 2. (c) The 68 transmural segments involved in step 3. (d) The additional three layers utilized in step 4. Note that at step 4 the layers are for each segment, bringing total number of subsegments to 204.

In the first step, the optimization procedure commenced with the simulation on the LV mesh (see Fig 5a), where we set *D*_initial_ = 0% of fibrosis. Subsequently, utilizing the interpolated LAT values, the algorithm computed a new fibrosis density using a minimization method (see the minimization algorithm described later). Finally, the optimization continued until an optimal fibrosis density for the entire LV (*D*_LV_) was achieved.

In the second step, we applied the same optimization procedure on the 17 transmural segments (see Fig 5b). However, in this step, the algorithm started from *D*_*LV*_ and optimized the fibrosis density (*D* = *D*_1_…*D*_17_) in each segment separately using the LAT from the segment’ surface. As a result, we obtained a LV mesh with an optimal fibrosis density in every of the 17 transmural segments.

In the third step, we further refined our estimates by subdividing each of the 17 segments into four parts and repeated the procedure for the resulting 68 segments, see Fig 5c. This enabled us to obtain an estimate of *D* = *D*_1_…*D*_68_ for each of the 68 transmural segments of the heart.

In the final step, we divided each of the 68 transmural segments into three layers: endocardial, epicardial, and mid-myocardial, see Fig 5d. This division resulted in a total of 204 segments within the LV. For each of the 68 segments, our goal was to achieve the minimal value of *PtP*^*endo*^ by adjusting *D*^*endo*^ within the endocardial section, the minimal *PtP*^*epi*^ by adjusting *D*^*epi*^ within the epicardial section, and the minimal *LAT* by adjusting *D*^*mid*^ within the mid-myocardial section. The optimization process for each segment occurred sequentially, with adjustments to *D*^*mid*^ potentially influencing *PtP*^*endo*^ and *PtP*^*epi*^, thereby necessitating an update of the optimal values of *D*^*endo*^ and *D*^*epi*^. Therefore, this iterative sequence was repeated until the convergence criterion was satisfied (see the minimization algorithm described later).

We applied the optimization procedure for groups of segments in parallel to segments that were not neighbours of each other, see Fig 1a and 1b. We selected the following clusters: group №1 (1, 3, 5, 14, 16), group №2 (2, 4, 6, 13, 15), group №3 (7, 9, 11, 17), and group №4 (8, 10, 12). Since we divided each of the 17 segments into 4 parts, we optimized only one of the 4 segments simultaneously. For example, for group №1, we first optimized the segments with number 1.1, 3.1, 5.1, 14.1, 16.1. Then, we optimized the segments 1.2, 3.2, 5.2, 14.2, 16.2, and so on.

#### Minimization algorithm

**Fig 6:**
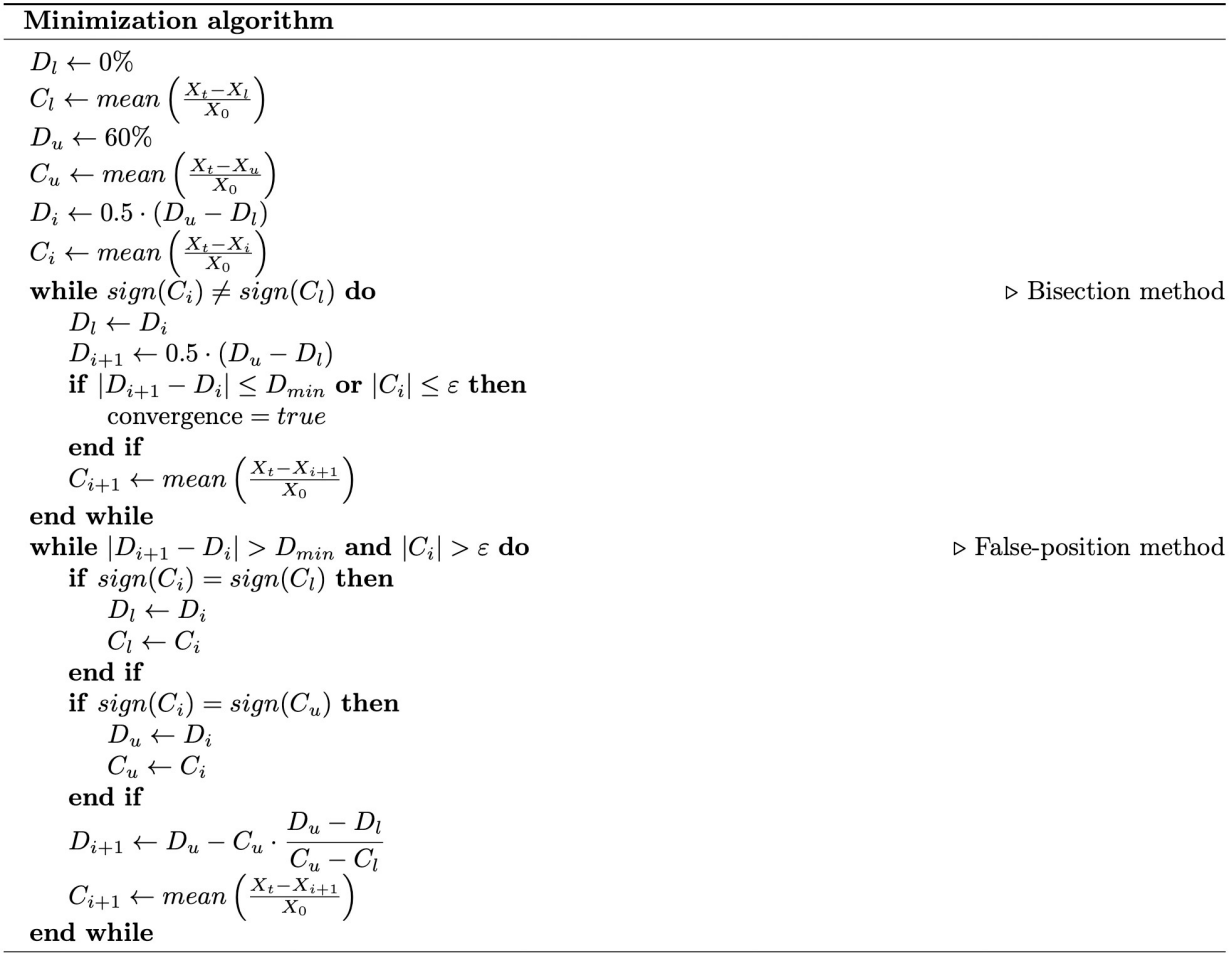
Minimization algorithm.

As described above, to minimize the LAT and the PtP, we utilized an adaptive minimization algorithm during each global and local optimization step, as illustrated in the pipeline in Fig 4. We will elucidate this algorithm step by step, which is summarized in Fig 6.

The minimization algorithm combines the bisection and the modified false-position method [34]. In basic terms, both methods necessitate sign-changing intervals for the cost (or objective) function. However, the bisection method solely requires a sign of the cost function values at both ends of the searching interval. Conversely, the false-position method utilizes the exact cost function values, thereby facilitating faster convergence.

We constructed a cost function *C*_*i*_ for both the LAT and PtP by averaging the residuals between the target *X*_*t*_ and the estimated *X*_*i*_, normalized by *X*_0_ (the shortest LAT and maximum PtP):

$$\begin{array}{c} C_{i}=mean(\frac{X_{t}-X_{i}}{X_{0}})=\frac{1}{N}\sum_{n=1}^{N}\frac{x_{n}^{t}-x_{n}^{i}}{x_{n}^{0}}, \end{array}$$ (4)

where $X_{t}=\left\{x_{0}^{t},x_{1}^{t},..,x_{N}^{t}\right\}$ is vector of target values (LAT or PtP), $X_{i}=\left\{x_{0}^{i},x_{1}^{i},..,x_{N}^{i}\right\}$ is vector of values at step *i*, $X_{0}=\left\{x_{0}^{0},x_{1}^{0},..,x_{N}^{0}\right\}$ is vector of values for 0% of fibrosis and *N* is number of points on the surface. Normalization of the LAT and PtP data is essential to eliminate the influence of confounding factors such as LV wall thickness and localized variations that could significantly alter the magnitudes of these parameters. This normalization ensures a more accurate assessment of fibrosis’s effect on EGM properties. Additionally, computing the mean of the normalized errors enhances the method’s robustness against potential measurement errors at specific points.

In 3D myocardium, wave propagation interruption occurs when fibrosis exceeds 60% [35, 36], resulting in an infinitely long LAT and a PtP of zero. Consequently, the exact value of the cost function is indeterminate at the upper limit of the interval [0%–60%]. To address this issue, we employed the bisection method, assuming that the cost function changes sign in the interval [0%–60%]. Initially, we computed the cost function value *C*_1_ for 0% fibrosis and assumed that the sign of the cost function value *C*_2_ for 60% fibrosis would be opposite. Following the bisection method, the next value *C*_3_ should lie at half the interval (30% fibrosis). If the sign of *C*_3_ is opposite to *C*_1_, the algorithm switches to the false-position method since we have both cost function values. Conversely, if the sign of *C*_3_ is the same as *C*_1_, then the bisection method continues for [30%–60%]. The iterations stop when the cost function becomes lower than the error tolerance value *ε* = 0.01:

$$\begin{array}{c} |\frac{1}{N}\sum_{n=1}^{N}\frac{x_{n}^{t}-x_{n}^{i}}{x_{n}^{0}}|<\varepsilon , \end{array}$$ (5)

or the density change is less than *D*_*min*_ = 1%:

$$\begin{array}{c} |D_{i+1}-D_{i}|<D_{min}, \end{array}$$ (6)

where *D*_*i*_ is the fibrosis density at step *i*, and *D*_*min*_ minimal allowed density change.

## Results

### Application of algorithm to four artificial models of fibrosis

**Fig 7:**
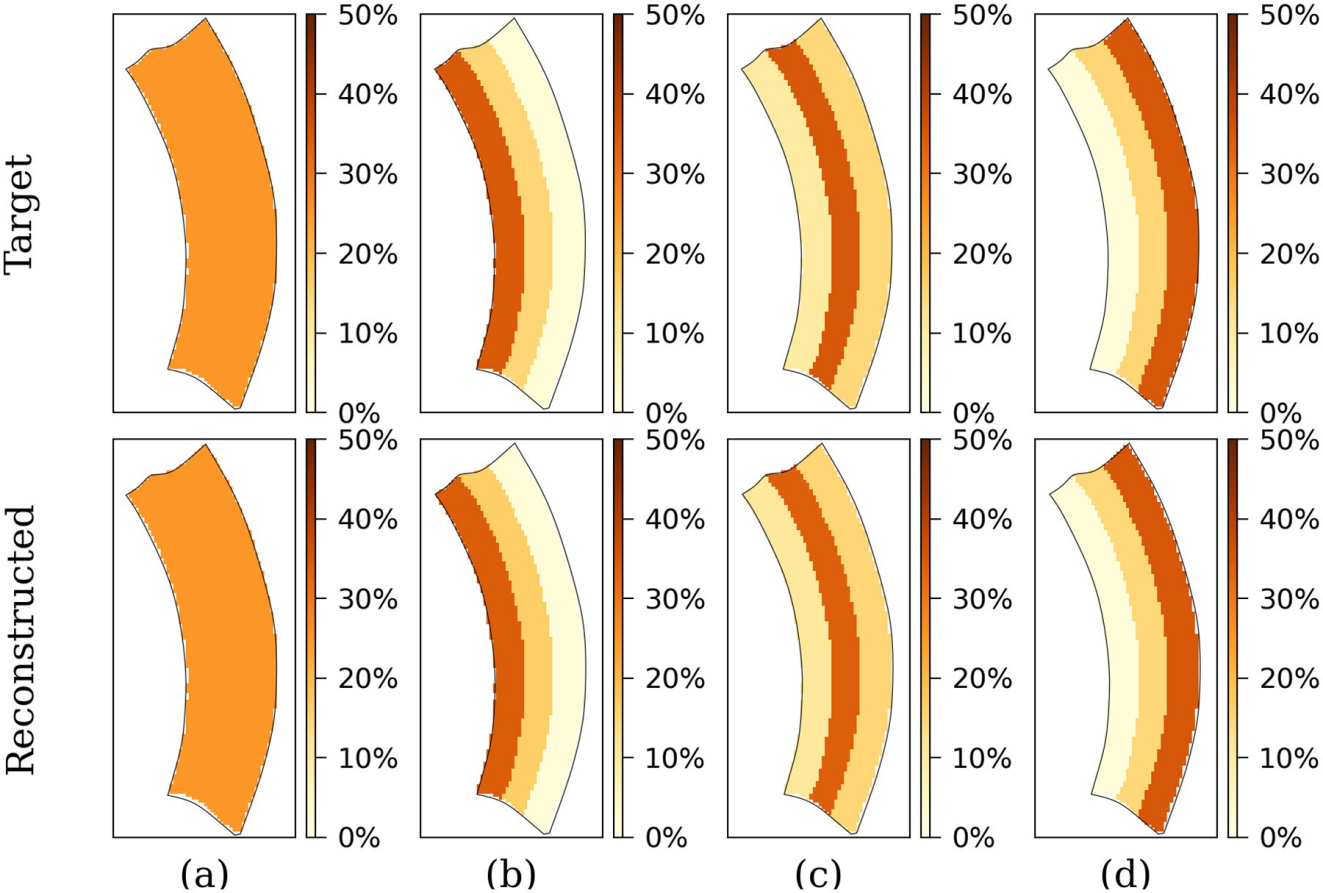
Comparison between the target fibrosis distributions and the results of the algorithm. The XY slices of the segment show the density within each layer of the segment No. 12 with: (a) transmural, (b) sub-endocardial, (c) mid-myocardial, and (d) sub-epicardial fibrosis.

Fig 7 shows the algorithm’s performance across four fibrosis patterns, with detailed error data in Table 2. In all cases, the algorithm accurately identified fibrotic regions. The local (per layer) error was negligible, remaining under 0.2% for homogeneous transmural and sub-epicardial fibrosis. However, sub-endocardial fibrosis yielded higher errors, notably reaching 1.4% in the mid-myocardial section. Although mid-myocardial fibrosis detection also succeeded, it resulted in errors of 1.4% (mid-myocard) and 0.8% (sub-endocardial).

**Table 2:** Absolute error in fibrosis density between target fibrosis and results of the algorithm for four patterns.

Patterns	Location in the heart wall		
	Sub-endocard	Mid-myocard	Sub-epicard
Transmural	0.1%	0.1%	0.2%
Sub-endocardial	0.5%	1.4%	0.4%
Mid-myocardial	0.8%	1.4%	0.4%
Sub-epicardial	0.1%	0.3%	0.1%

**Fig 8:**
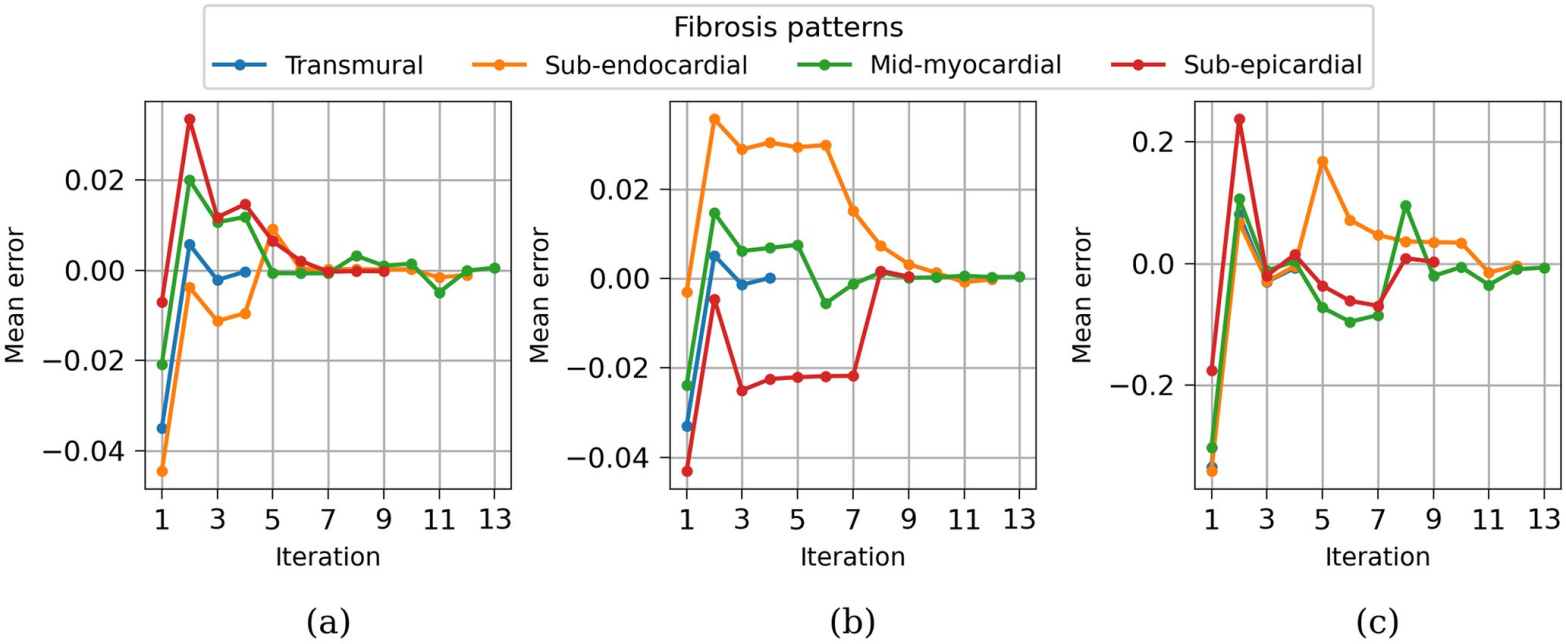
Convergence of the algorithm for four idealized fibrosis patterns. (a) Convergence of endocardial PtP. (b) Convergence of epicardial PtP. (c) Convergence of LAT.

Fig 8 illustrates the convergence graphs for all studied cases. It is evident that the convergence is remarkably rapid for transmural fibrosis (represented by the blue line), with the desired result achieved after only four iterations. In contrast, for other locations of fibrosis, convergence requires approximately 8-12 iterations, with the convergence of the LAT occurring faster than that of the EGM amplitude.

### Application of algorithm to the fifth accurate model of fibrosis

**Fig 9:**
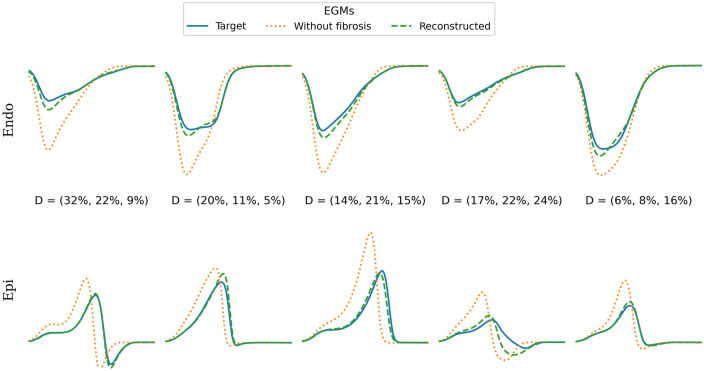
Representation of endocardial and epicardial electrograms across five spatial locations in a 68-segment LV model. Each column corresponds to a specific segment, denoting the fibrosis density in parentheses for endocardial, mid-myocardial, and epicardial layers.

We applied our approach to analyze experimental data on fibrosis in a patient’s heart with NICM. Fig 9 presents representative examples of EGMs at five spatial locations. The orange line depicts EGMs in the model without fibrosis, while the blue line represents the target EGMs and optimized EGMs obtained by our algorithm. Our method accurately reconstructs the target EGMs, particularly reproducing the PtP of the EGM with good accuracy. However, it is worth noting that simulated EGMs do not exhibit fractionation. We address this aspect in the discussion section.

**Fig 10:**
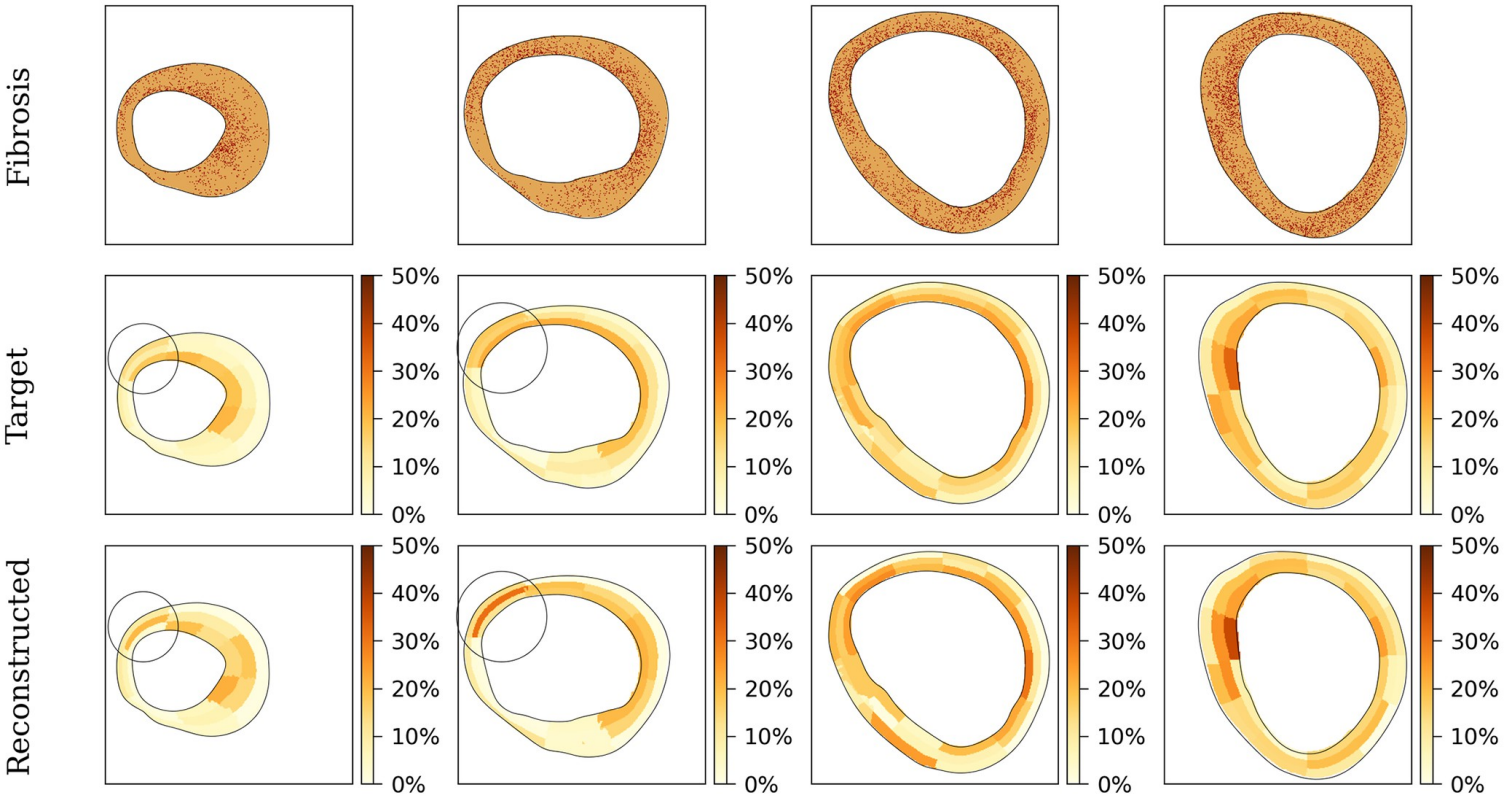
Comparison between the target fibrosis distributions and the outputs of the algorithm. Four different XY slices of the left ventricle are shown, with highlighted areas indicating segments with significant errors between the target density distribution and algorithm estimations.

Our primary focus is on accurately reconstructing the spatial distribution and pattern of fibrosis. Fig 10 (top) displays four LV slices with fibrosis. The “target” depicts the fibrosis densities obtained from the patient’s data, while the “reconstructed” shows the results of the optimization algorithm. We found that the reconstructed data closely resemble the target data and capture the qualitative fibrosis distribution well. In most sections, the algorithm accurately identifies areas of higher fibrosis. The patterns in the two right columns of Fig 10 are examples of a good match. However, in some cases, we can visually identify errors in the percentage of fibrosis. For instance, in the first left column of Fig 10, there is a discrepancy in the estimation of sub-endocardial fibrosis in the highlighted area. While the target data show the largest fibrosis area located at the endocardial surface, in the estimated data, in addition to fibrosis at the sub-endocardial area, we also observe some fibrosis at the sub-epicardial area and a larger extent of fibrosis in the mid-myocardium. A similar segment can be identified in the second column as well. In both cases, we observed higher mid-myocardium fibrosis in the estimated data compared to the target data.

**Fig 11:**
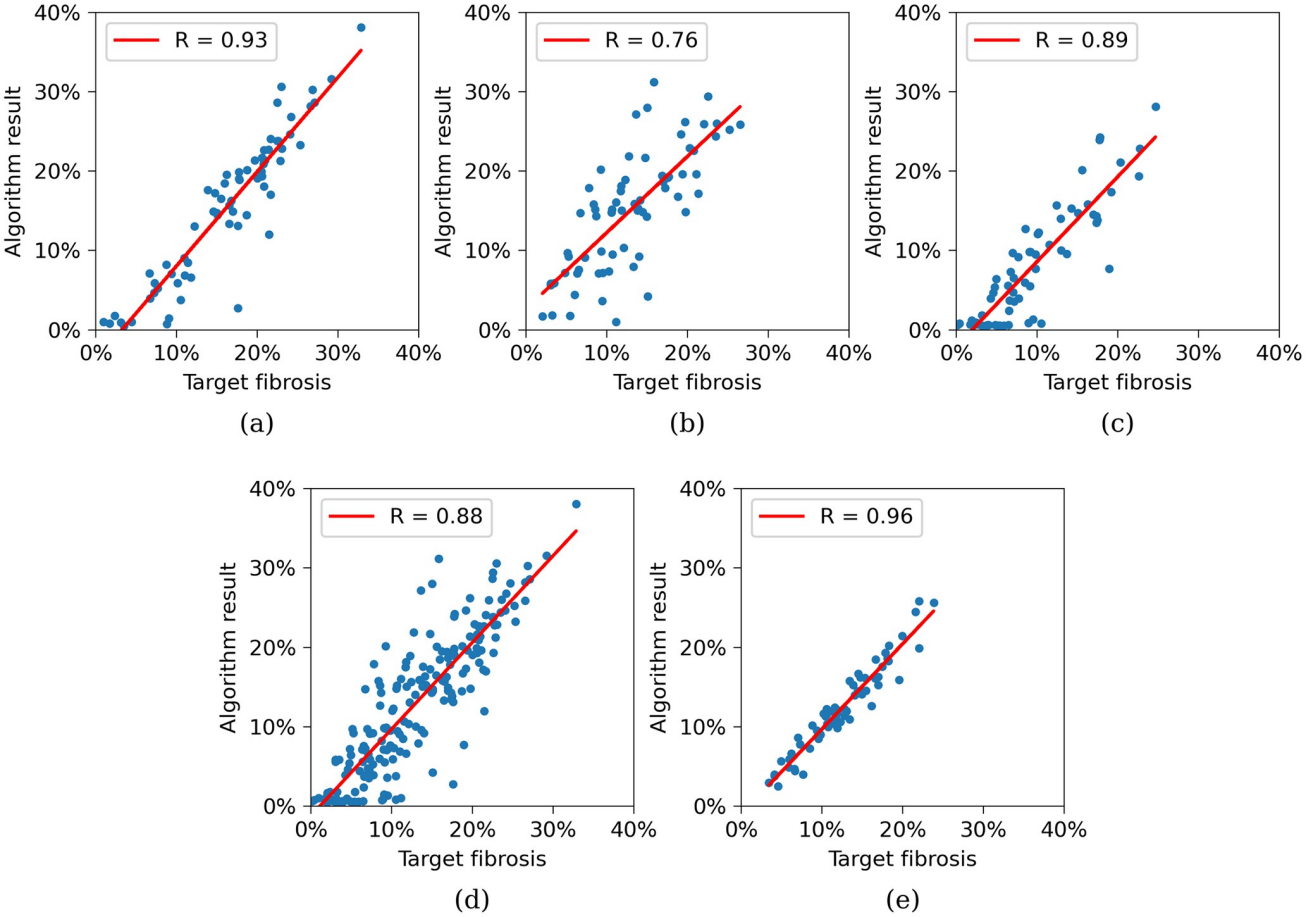
Correlation between target fibrosis densities and outputs of the algorithm. (a) Correlation for 68 sub-endocardial segments. (b) Correlation for 68 mid-myocardial segments. (c) Correlation for 68 sub-epicardial segments. (d) Correlation for all 204 segments. (e) Correlation for average density in 68 transmural segments.

To further quantify the quality of our estimations, we plotted the target and estimated percentages of fibrosis for groups of segments in Fig 11. We observed a linear correlation for 68 sub-endocardial (a), 68 mid-myocardial (b), and 68 sub-epicardial segments (c). The sub-endocardial segments exhibited the best result, with a correlation coefficient of 0.93, while the mid-myocardial segments showed the lowest correlation coefficient of 0.76. We also assessed the correlation for all 204 segments (d), yielding a correlation coefficient of 0.88 with a regression line slope close to the ideal case of 1 (1.09). Additionally, we examined the correlation for the 68 transmural segments (e) (segments not divided into three layers), obtaining a correlation coefficient of 0.96, which is closest among all groups to the ideal value of 1. This is expected, as it is calculated for a larger, less fragmented segment.

In addition, we calculated the density estimation errors for all 204 segments and analyzed them by the same groups, as shown in Table 3. Consistent with our expectations, the maximum error was observed for mid-myocardial segments, with a mean of 2.1% and a standard deviation of 4.9%. The mean error across all 204 segments was 0%, with a standard deviation of 4.3%. For the transmural segments, the mean error was 0.1%, with a deviation of 1.5%. Thus, our method accurately determines the average amount of fibrosis transmurally. However, it slightly overestimates density in mid-myocardial segments.

**Table 3:** The fibrosis density estimation error for distinct group of segments.

Segments	*N*	Correlation		Absolute error	
		*R*	slope	mean	std
Sub-endocardial	68	0.93	1.19	0.8%	3.6%
Mid-myocard	68	0.76	0.96	-2.1%	4.9%
Sub-epicardial	68	0.89	1.07	1.4%	3.2%
All	204	0.88	1.09	0.0%	4.3%
Transmural	68	0.96	1.07	0.1%	1.5%

In our LV model, among the 204 segments, 39 segments have more than 21% of fibrosis, which can be considered abnormal [14]. We effectively localized 31 of them: 20 out of 25 in the endocardium, 3 out of 4 in the epicardium, and 8 out of 10 in the mid-myocardial region.

**Fig 12:**
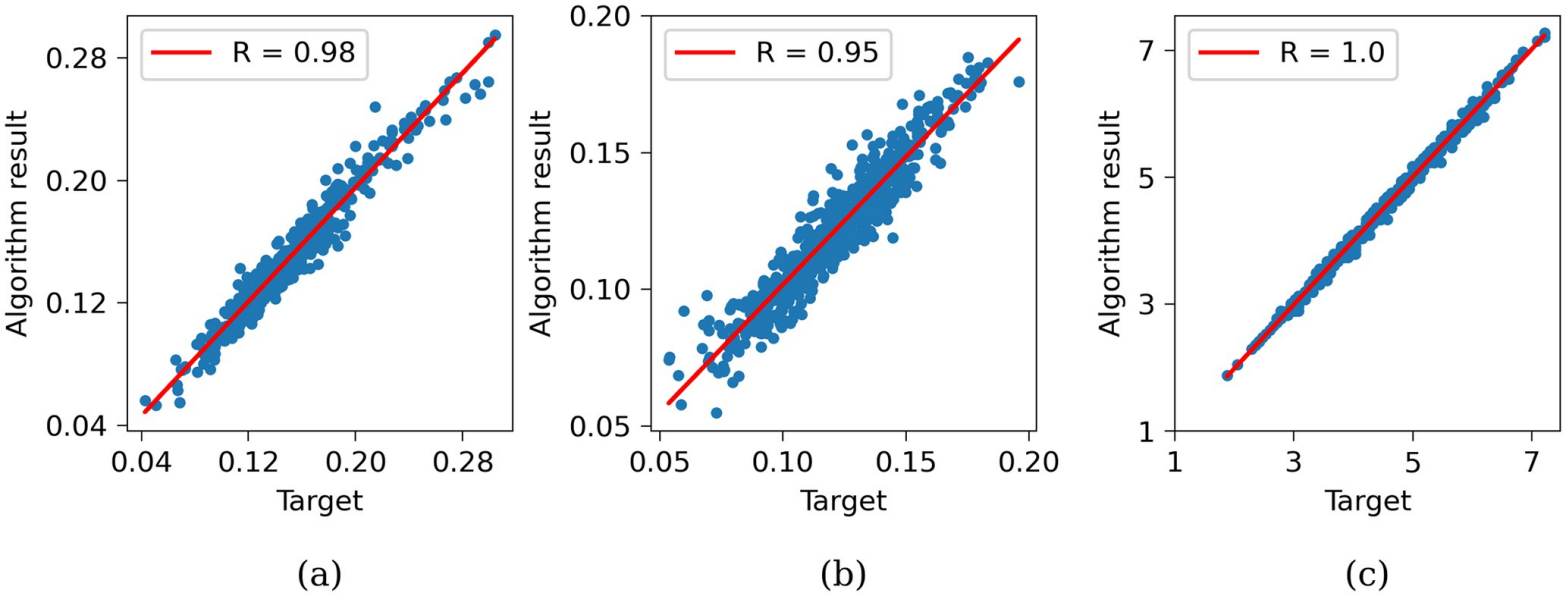
Correlation between target and algorithm-reproduced values. (a) Endocardial PtP correlation. (b) Epicardial PtP correlation. (c) LAT correlation.

Fig 12 displays distributions of LAT and PtP for both the target model and the output of the algorithm. We observed a linear relationship between the target and estimated data. Specifically, the correlation line for LAT was almost ideal (*R* = 1.0), while the correlation for endocardial PtP was 0.98, and for epicardial PtP was 0.95. A higher correlation for LAT can be expected because we minimize it during multiscale optimization and keep it small in the local per-segment optimization procedure. Also, the LAT at each segment is less dependent on the neighboring segments, while the amplitudes can be affected by remote myocardial bundles.

**Fig 13:**
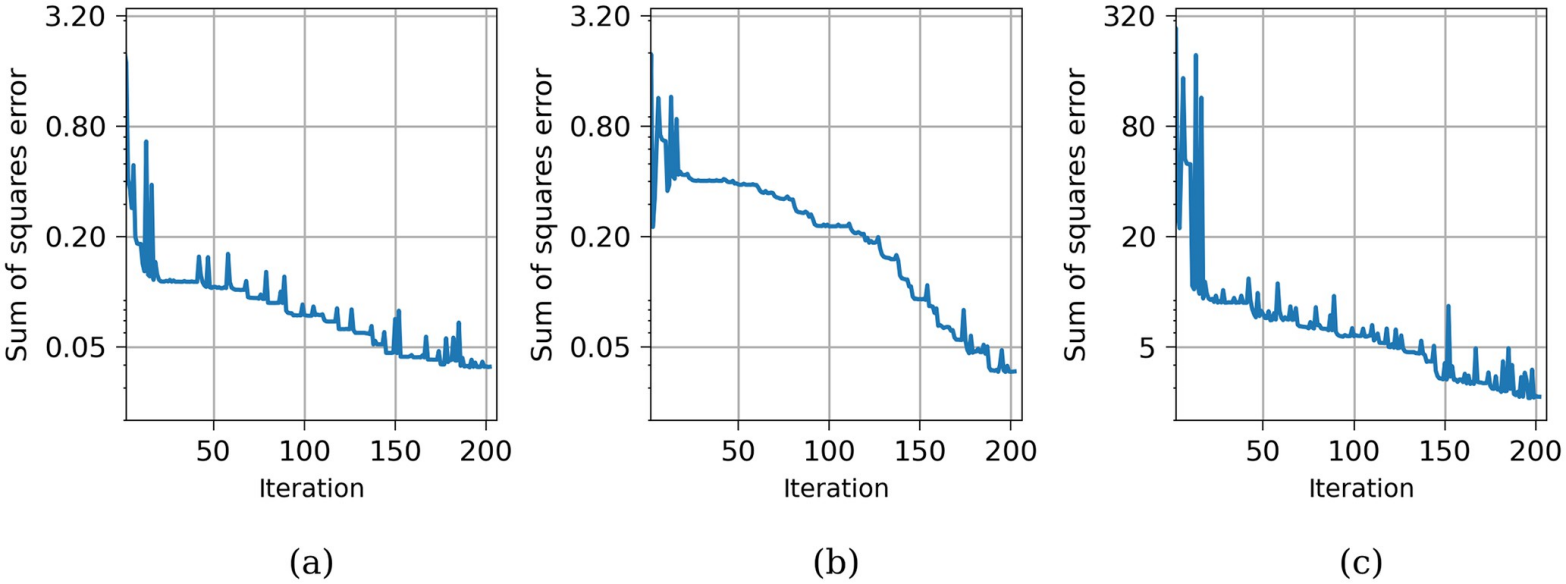
Convergence of the algorithm for the entire left ventricle. (a) Convergence of endocardial PtP. (b) Convergence of epicardial PtP. (c) Convergence of LAT.

Fig 13 illustrates the convergence process of the method for estimating the density throughout the entire LV. The optimization procedure employs a sequential minimization method, which may entail numerous iterations, increasing proportionately to the number of segments. However, by implementing a concurrent computation approach for distant segments (as elaborated in the Methods section), we achieved the desired result in just 201 iterations. The concurrent computation is possible because the influence of remote myocardium on PtP amplitudes diminishes at a factor of two in terms of distance, while LAT values are primarily determined by the tissue within the respective segment itself.

### Arrhythmia initiation

The primary objective of any substrate study is to pinpoint regions where arrhythmia initiation can occur, subsequently targeted for ablation. Thus, we endeavored to induce arrhythmia through high-frequency pacing in both the target and reconstructed models, comparing the outcomes. We successfully initiated a spiral wave at a time step of 28.5 model time units in both the original and reconstructed meshes. Additional technical specifics are detailed in the methods section.

**Fig 14:**
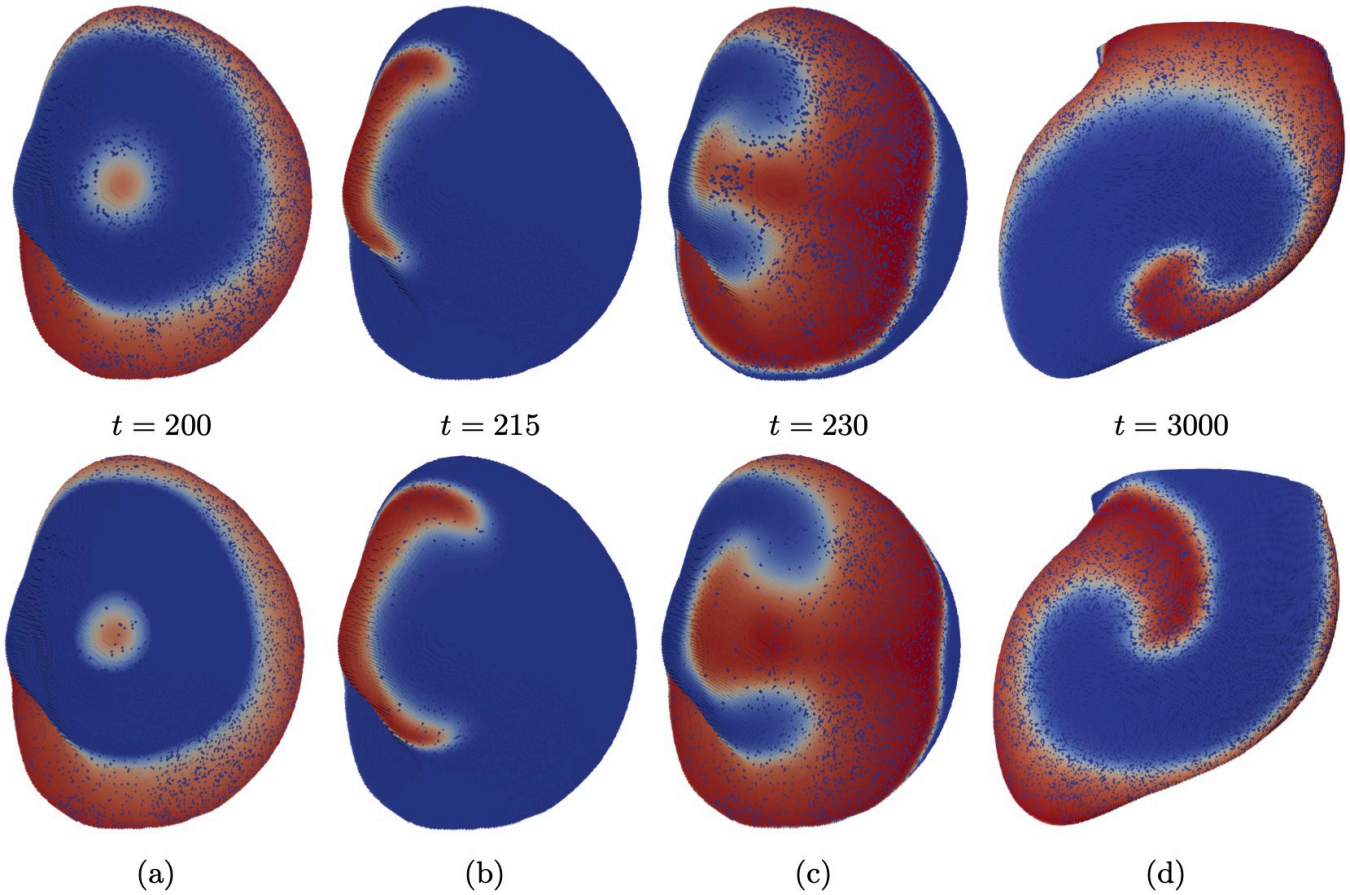
Induction of arrhythmia for the target fibrosis model (upper) and output of the algorithm (lower). (a) Stimulation at time 200. (b) Wave breakups. (c) Initiation of spiral waves. (d) Stabilized spiral waves.

In both datasets, the presence of fibrosis in the lateral region of the LV resulted in wave breakup, as illustrated in Fig 14b. In both models, wave breakage occurred at approximately the same location and exhibited a similar excitation pattern. Consequently, the wave re-entered the fibrotic region, initiating two spiral waves, as depicted in Fig 14c. Once again, the excitation patterns in both datasets are comparable. Subsequently, in both datasets, one of the spiral waves dissipated at the heart’s boundary, while the other stabilized in the fibrotic region (refer to Fig 14d). We once again observe similar excitation patterns, albeit with a slight phase shift due to differences in propagation velocity, resulting in a phase shift over time.

**Fig 15:**
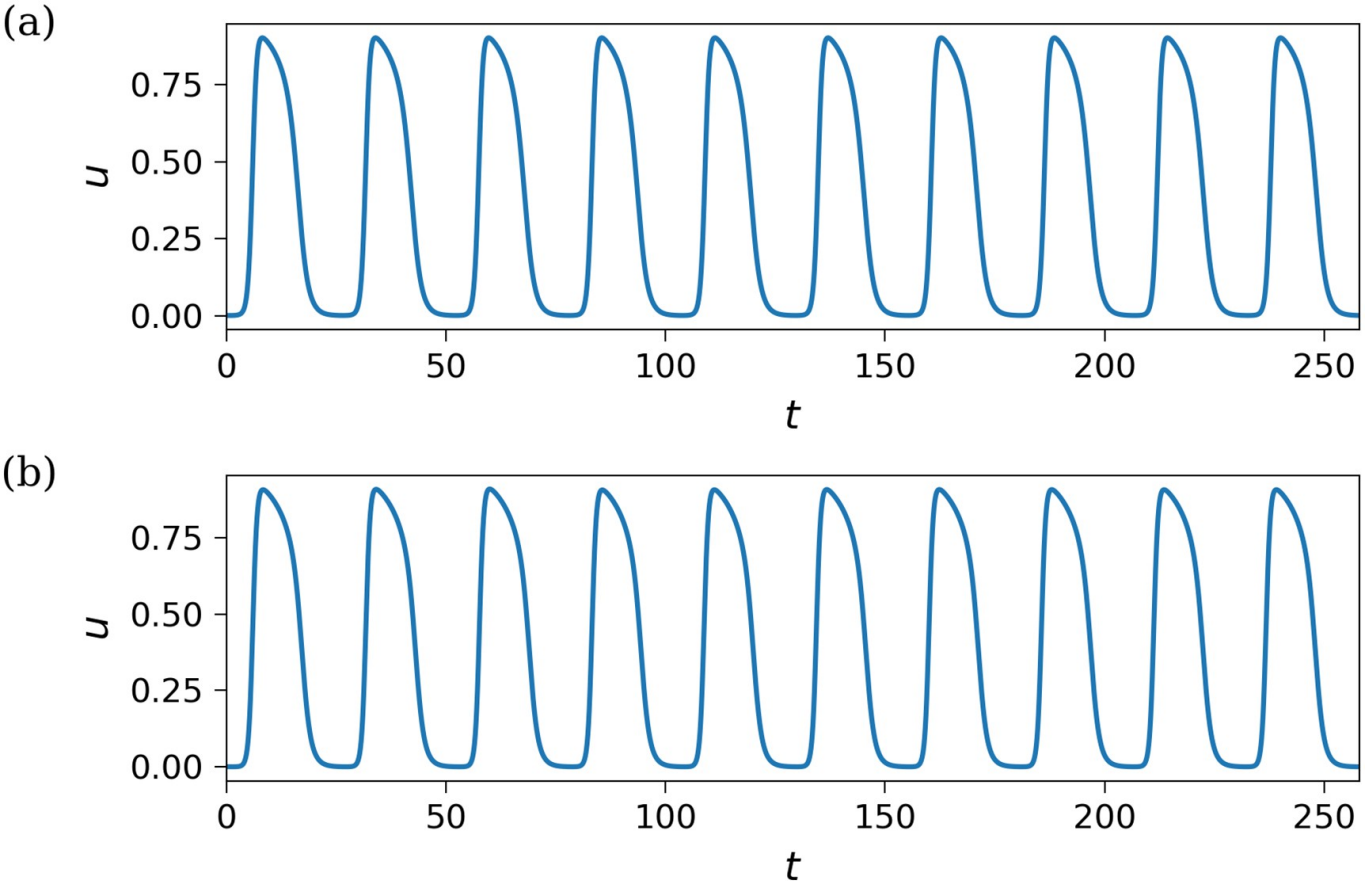
Transmembrane potential recorded at a point located outside the core of the spiral wave for. (a) The target fibrosis model. (b) The output of the algorithm.

Once the spiral wave stabilized, we assessed the transmembrane potential at a point outside the wave’s core. We determined the period as the time interval between the peaks of the transmembrane potential, averaged over ten cycles (see Fig 15). Since the periods and locations of the arrhythmia in the original mesh and the mesh after optimization are closely aligned (at 25.77 and 25.65 time units, respectively), we can infer that our algorithm effectively reconstructed the arrhythmogenic substrate in the LV.

## Discussion

This paper introduced an algorithm designed to reconstruct 3D fibrosis patterns within the left ventricle wall. The algorithm aims to minimize errors in LATs and PtPs of unipolar electrograms recorded on both endo- and epicardial surfaces during sinus rhythm.

The algorithm effectively detected all four idealized fibrosis patterns in test models with errors of less than 2%. Additionally, it optimized fibrosis density in 204 segments of the LV using patient-specific fibrosis generated from histological images. The mean error for the segment groups was as follows: for mid-myocardial fibrosis, −2.1 ± 4.9%; for sub-endocardial fibrosis, 0.8 ± 3.6%; and for sub-epicardial fibrosis, 1.4 ± 3.2%. Moreover, the algorithm successfully localized 31 out of 39 segments with high-density fibrosis (> 21%), typically targeted for ablation. Furthermore, inducing arrhythmia through high-frequency pacing of both “target” and “reconstructed” LV models resulted in stabilized spiral waves on the same side of the LV, with periods exhibiting a difference of less than 0.5% (25.77 vs 25.65). These results underscore the method’s potential for accurately identifying the fibrosis content of the heart.

Our method evolved through several stages. Initially, we attempted to reconstruct fibrosis solely by minimizing LAT. In previous work [18, 19], the authors proposed using LAT to determine the conduction properties of cardiac tissue. They optimized a 2D effective diffusion coefficient (apparent conductivity) to locate areas of slow conduction in simulated and clinical data. However, when we applied this approach to the entire 3D problem, we found that it struggled to determine the location of intramural fibrosis under normal cardiac excitation, where the wave propagated from the endocardial to the epicardial surface. This was because patterns with mid-myocardial, sub-endocardial, or sub-epicardial fibrosis locations could lead to similar LAT values.

To address this challenge, we incorporated the PtP amplitude of EGMs as additional parameters for optimization. In clinical practice, unipolar and bipolar PtPs with varying cut-off values are commonly used as indicators of fibrosis [13, 21]. However, in NICM patients with ventricular tachycardias, these cut-off values often perform poorly [14]. Furthermore, relying solely on the amplitude of the EGM can make it difficult to distinguish whether the potential originates from a nearby pathological region or a normally excited distant region.

By integrating PtP amplitudes and LAT values from both endocardial and epicardial EGMs, we notably improved the precision of fibrosis localization within the myocardial wall. Our approach effectively identified mid-myocardial fibrosis in 8 out of 10 cases, a traditionally challenging task using electrograms alone. This underscores the crucial role of combining PtP and LAT measurements in our algorithm, enhancing its effectiveness in accurately detecting mid-myocardial fibrosis.

One limitation of our study is that we relied on data from patients with NICM without dense scars and identifiable isthmuses. Ablating NICM hearts can be challenging due to the limitations of non-invasive methods like LGE-CMR in quantifying fibrosis and the challenges in applying cut-off values from electroanatomical voltage mapping. Our method primarily addresses fibrosis determination in such scenarios. However, it has the potential to be adapted for patients with other pathologies, including myocardial infarction. Nevertheless, further research and modifications are necessary to validate its applicability in those contexts.

Another notable observation is that the simulated EGMs (Fig 9) exhibit a reduction in PtP near the fibrotic area. However, they do not display significant fractionation, typically observed in regions with extensive scar transmurality and substantial patchy fibrosis [37]. This could be attributed to the utilization of a low-dimensional model for cardiac excitation. In such models, the spatial length of the wavefront is longer compared to ionic models, making it less likely for front disturbances due to fibrosis to translate into EGM fractionation. Nevertheless, this approach accurately reproduces integral EGM characteristics, such as changes in amplitude due to the presence of unexcitable regions. Since our study primarily relies on PtP and LAT as the main criterion for substrate identification, this approach suffices. However, exploring whether other EGM characteristics, including fractionation, can enhance substrate characterization would be an interesting avenue for future research.

The algorithm relies on simultaneous endocardial and epicardial recordings, along with concurrent excitation of the endocardial surface. However, these assumptions do not hold for the septal area of the heart. Firstly, under sinus rhythm, septal excitation does not occur synchronously due to the anatomy of the conduction system [38]. Secondly, during ventricular activation, both sides of the septum are excited via the Purkinje system. Additionally, other pathological conditions, such as myocardial infarction or NICM, can also alter the endocardial excitation pattern. In such cases, actual endocardial activation maps could be used to adjust the algorithm’s input. Therefore, enhancing the algorithm for clinical settings is crucial for obtaining reliable results.

Our method holds promise for integration with non-invasive mapping techniques, such as those enabling the reconstruction of potentials on the epicardial surface through the inverse ECG problem [39]. Additionally, a similar method for non-invasive inverse mapping of the endocardial surface is currently in development [40]. Consequently, our approach could potentially be expanded to leverage such non-invasive recordings, broadening its applicability and enhancing its clinical utility.

While our study utilized a two-variable Aliev-Panfilov model as the basis for our electrophysiological simulations, employing more complex ionic models tailored for human cardiac tissue could potentially improve accuracy, albeit at the cost of longer computational time [41]. Alternatively, more computationally efficient eikonal models for wave propagation [42] could be explored, although they may not fully capture action potential repolarization dynamics.

From a technical standpoint, there are avenues for refining the method. For example, increasing the number of segments could enhance accuracy. While our study relied on the standard 17-segment model as a basis, subdividing each segment into four equal parts with three transmurally divided layers, expanding the segmentation further would lead to higher computational expenses. Such an expansion would require a better grasp of the electrode “field of view” and potentially modifying the algorithm to integrate weighted input from EGM and LAT. While this could significantly improve reconstruction accuracy, it would be intriguing to consider reconstructing a continuous fibrosis distribution across the heart rather than a constant value for each segment. This continuous approach could provide new insights.

Our paper should be viewed as an initial step in a novel methodology that holds potential for clinical application. We demonstrate the feasibility of using mathematical tools to recover the spatial distribution of fibrosis within the ventricular walls from EGM recordings without relying on voltage cut-off values. We envision that with further exploration, such an approach or a similar one could eventually be employed independently or in conjunction with existing methods to potentially improve accuracy, particularly in assessing fibrosis within the heart wall. Moreover, considering patient-specific geometry and incorporating more detailed patient data could enhance the model’s clinical relevance. In essence, our study lays the groundwork for future investigations in these directions.
